# The Association Between Arterial Oxygen Level and Outcome in Neurocritically Ill Patients is not Affected by Blood Pressure

**DOI:** 10.1007/s12028-020-01178-w

**Published:** 2021-01-05

**Authors:** Jaana Humaloja, Markus B. Skrifvars, Rahul Raj, Erika Wilkman, Pirkka T. Pekkarinen, Stepani Bendel, Matti Reinikainen, Erik Litonius

**Affiliations:** 1 Department of Emergency Care and Services, University of Helsinki and Helsinki University Hospital, Helsinki, Finland; 2 Department of Neurosurgery, University of Helsinki and Helsinki University Hospital, Helsinki, Finland; 3 Division of Intensive Care Medicine, Department of Anesthesiology, Intensive Care and Pain Medicine, University of Helsinki and Helsinki University Hospital, Helsinki, Finland; 4 Department of Anesthesiology and Intensive Care, Kuopio University Hospital & University of Eastern Finland, Kuopio, Finland; 5 Division of Anesthesiology, Department of Anesthesiology, Intensive Care and Pain Medicine, University of Helsinki and Helsinki University Hospital, Helsinki, Finland

**Keywords:** Traumatic brain injury, Cardiac arrest, Subarachnoid hemorrhage, Intracranial hemorrhage, Acute ischemic stroke, Hypotension, Partial pressure of arterial oxygen, Hyperoxemia

## Abstract

**Background:**

In neurocritically ill patients, one early mechanism behind secondary brain injury is low systemic blood pressure resulting in inadequate cerebral perfusion and consequent hypoxia. Intuitively, higher partial pressures of arterial oxygen (PaO_2_) could be protective in case of inadequate cerebral circulation related to hemodynamic instability.

**Study purpose:**

We examined whether the association between PaO_2_ and mortality is different in patients with low compared to normal and high mean arterial pressure (MAP) in patients after various types of brain injury.

**Methods:**

We screened the Finnish Intensive Care Consortium database for mechanically ventilated adult (≥ 18) brain injury patients treated in several tertiary intensive care units (ICUs) between 2003 and 2013. Admission diagnoses included traumatic brain injury, cardiac arrest, subarachnoid and intracranial hemorrhage, and acute ischemic stroke. The primary exposures of interest were PaO_2_ (recorded in connection with the lowest measured PaO_2_/fraction of inspired oxygen ratio) and the lowest MAP, recorded during the first 24 h in the ICU. PaO_2_ was grouped as follows: hypoxemia (< 8.2 kPa, the lowest 10th percentile), normoxemia (8.2–18.3 kPa), and hyperoxemia (> 18.3 kPa, the highest 10th percentile), and MAP was divided into equally sized tertiles (< 60, 60–68, and > 68 mmHg). The primary outcome was 1-year mortality. We tested the association between hyperoxemia, MAP, and mortality with a multivariable logistic regression model, including the PaO_2_, MAP, and interaction of PaO_2_*MAP, adjusting for age, admission diagnosis, premorbid physical performance, vasoactive use, intracranial pressure monitoring use, and disease severity. The relationship between predicted 1-year mortality and PaO_2_ was visualized with locally weighted scatterplot smoothing curves (Loess) for different MAP levels.

**Results:**

From a total of 8290 patients, 3912 (47%) were dead at 1 year. PaO_2_ was not an independent predictor of mortality: the odds ratio (OR) for hyperoxemia was 1.16 (95% CI 0.85–1.59) and for hypoxemia 1.24 (95% CI 0.96–1.61) compared to normoxemia. Higher MAP predicted lower mortality: OR for MAP 60–68 mmHg was 0.73 (95% CI 0.64–0.84) and for MAP > 68 mmHg 0.80 (95% CI 0.69–0.92) compared to MAP < 60 mmHg. The interaction term PaO_2_*MAP was nonsignificant. In Loess visualization, the relationship between PaO_2_ and predicted mortality appeared similar in all MAP tertiles.

**Conclusions:**

During the first 24 h of ICU treatment in mechanically ventilated brain injured patients, the association between PaO_2_ and mortality was not different in patients with low compared to normal MAP.

**Supplementary Information:**

The online version of this article (10.1007/s12028-020-01178-w) contains supplementary material, which is available to authorized users.

## Introduction

Brain ischemia is an important, and possibly modifiable, mechanism of secondary brain injury in patients with various types of neurocritical illness [1, 2]. For this reason, critically ill patients with brain injury frequently receive supplemental oxygen with the hope of increasing oxygen supply to the compromised parts of the brain. Oxygen therapy should be carefully titrated, as hyperoxemia has been associated with worse outcomes in some clinical studies of patients with traumatic brain injury (TBI) or cerebrovascular insult and in patients after cardiac arrest (CA) [3–6]. Possible mechanisms include an increased formation of reactive oxygen species (ROS), alterations to brain metabolic function, hyperoxia-induced vasoconstriction, and damage to non-injured parts of the brain [7, 8]. The evidence is conflicting, with some studies suggesting lack of harm from hyperoxemia exposure and some studies even suggest benefit [9–11].

During circulatory shock brain perfusion decreases resulting in impaired tissue oxygenation and possibly worsening brain ischemia. As both arterial oxygen content and mean arterial pressure (MAP) influence brain tissue oxygenation, it appears intuitive that brain ischemia related to decreased perfusion during hypotension could be alleviated by intermittently targeting higher oxygen levels in blood [12]. In the current study, we aimed to determine the association between arterial oxygen tension (PaO_2_) and long-term outcome in brain injury patients with various severity of hemodynamic instability and low blood pressure during the initial 24 h of ICU treatment. We hypothesized that even though hyperoxemia has been associated with poorer outcomes in several brain injury populations, it could offer protection especially among patients with hypotension and therefore likely insufficient brain perfusion. Accordingly, we designed a retrospective study of the association between arterial oxygen and outcome in patients with various types of brain injury and especially focused on studying the interactions between MAP and partial pressure of arterial blood oxygen (PaO_2_).

## Materials and Methods

### Study Setting and Data Sources

We retrospectively screened the Finnish Intensive Care Consortium (FICC) database for adult (age ≥ 18) patients admitted to five university-affiliated tertiary referral hospitals’ ICUs in Finland in the years 2003–2013 with various types of brain injury [13]. FICC coordinates an intensive care benchmarking program and a multicenter database that collects data from several Finnish ICUs; TietoEvry (Kuopio, Finland) maintains the central database. FICC was established in 1994, and the initial aim was to improve intensive care quality in Finland. We included patients with different types of brain injury: the admission diagnoses included TBI, subarachnoid hemorrhage (SAH), intracranial hemorrhage (ICH), acute ischemic stroke (AIS), and resuscitated CA. We defined the diagnoses of TBI, ICH, SAH, AIS, and CA according to the Acute Physiology and Chronic Health Evaluation III (APACHE III) and the International Classification of Diseases and Related Health Problems, 10th Revision [14, 15]. CA patients included both in-hospital and out-of-hospital arrests admitted to the ICU. We grouped ICH and AIS patients together due to the small number of participants in each group. We only included patients on mechanical ventilation support during the initial 24 h in the ICU. We excluded readmissions. All participating ICUs followed internationally recognized treatment guidelines pertaining at the time. The study included no interventions. Data about admission diagnosis, premorbid physical performance, arterial oxygen value (PaO_2_) and other physiological data, and Simplified Acute Physiology Score II (SAPS II) components and scores were collected from the FICC database [16, 17]. Data were extracted in September 2016. The study data have previously been used in two studies reporting healthcare-associated costs after various types of brain injury, and data have partially been used in a study reporting associations between hyperoxemia and mortality after TBI [9, 18, 19]. The ethics committee of the Operative Division of Helsinki University Hospital, the Finnish National Institute for Health and Welfare, and the Social Insurance Institution of Finland (Kela) approved the study.

### Study Variables

#### Outcome Measures

The primary outcome was 1-year mortality, and as a sensitivity analysis we also inspected 90-day mortality. The confirmed dates of any possible deaths were collected from the FICC database and the Finnish Population Register Centre database. Functional outcome 1 year after ICU admission was the secondary outcome. We defined permanent disability or death as an unfavorable functional outcome. We used a surrogate marker for permanent disability that was defined as if the patient was granted a permanent disability allowance or disability pension by the Social Insurance Institution of Finland (Kela) 1 year after the insult [18]. All Finnish residents belong to the public social insurance system and are entitled to disability allowance or pension if their need for aid in daily activities is permanent. We only included patients with independent premorbid functional status in the analysis concerning functional outcome. Premorbid physical performance was determined by a modified version of the World Health Organization/Eastern Co-operative Oncology (WHO/ECOG) classification used by the FICC; patients were included for the secondary analysis with scores less than three (capable of self-care) [20].

#### Oxygenation Variables and Mean Arterial Pressure

Arterial PaO_2_ was the primary exposure of interest. The FICC database contains only one PaO_2_ value, which is the PaO_2_ associated with the lowest PaO_2_/FiO_2_ ratio during the initial 24 h in ICU. We categorized PaO_2_ as follows: hypoxemia (PaO_2_ < 8.2 kPa), normoxemia (PaO_2_ 8.2 to 18.3 kPa), and hyperoxemia (PaO_2_ > 18.3 kPa). We chose cut-points for categories according to the highest and lowest 10th percentile of all PaO_2_ values in the study data, rounded to the nearest 0.1 decimal point. The second exposure of interest was the lowest value of mean arterial pressure (MAP) during the initial 24 h in the ICU. MAP was grouped into tertiles: < 60 mmHg, 60–68 mmHg, and > 68 mmHg.

### Statistical Analyses

Categorical data are presented as counts and percentages and continuous data as medians with interquartile ranges (IQR). For mortality prediction, we constructed a multivariable logistic regression model, including age, admission diagnosis, independent premorbid physical performance (fully capable in self-care/dependent on help), intracranial pressure (ICP) monitored (yes/no), any vasoactive used (yes/no), and severity of disease (a modified SAPS II score excluding points for age, type of admission, oxygenation, and systolic blood pressure). In this model, we included categorized PaO_2_, MAP tertiles, and their interaction term PaO_2_*MAP. We chose the confounders to the model by clinical relevance and used the variance inflation factor to control for multicollinearity. First, we analyzed all the diagnosis cohorts together in one multivariable regression model, and, second, we conducted sensitivity analyses with diagnosis cohorts separately (TBI, CA, SAH, and ICH + AIH). In the sensitivity analyses, the TBI model was further adjusted for the Marshall classification of TBI based on the results of the initial computer tomography scans [21]. The results from the regression analyses are presented as odds ratios (OR) with 95% confidence intervals (95% CI) and *p* values shown in tables. Additionally, we visualized 1-year mortality with Kaplan–Meier curves. We inspected functional outcome with multivariable logistic regression model similar as with mortality prediction but without the variable premorbid physical performance (analysis only included pre-admission independent patients). Last, we wanted to demonstrate the independent relationship between PaO_2_ and predicted probability of 1-year mortality in MAP tertiles in different diagnosis cohorts. We calculated the predicted probability of mortality with multivariable logistic regression analysis separately in every diagnosis cohort with the following variables: age, premorbid physical performance, ICP monitoring use, vasoactive use, modified SAPS II score, and TBI Marshall classification when applicable. We used locally weighted scatterplot smoothing (Loess) curves to visualize the relationship between PaO_2_ and the predicted probability of 1-year mortality. In the scatterplot, all PaO_2_ values > 40 kPa were coded as 40 kPa, to avoid few high values’ strong impact to the curve shape. We tested the model accuracy with all regression analyses with receiver operating characteristic (ROC) curves and determined the area under the curve (AUC). We used the Hosmer–Lemeshow Ĉ test to evaluate the model fit. Before proceeding with any statistical method, we ensured that the assumptions of the method in question were appropriately met. We performed all analyses with SPSS Statistics 25.0 for Mac OS (IBM Corp, Armonk, NY, USA).

## Results

### Baseline Characteristics

**Fig. 1:**
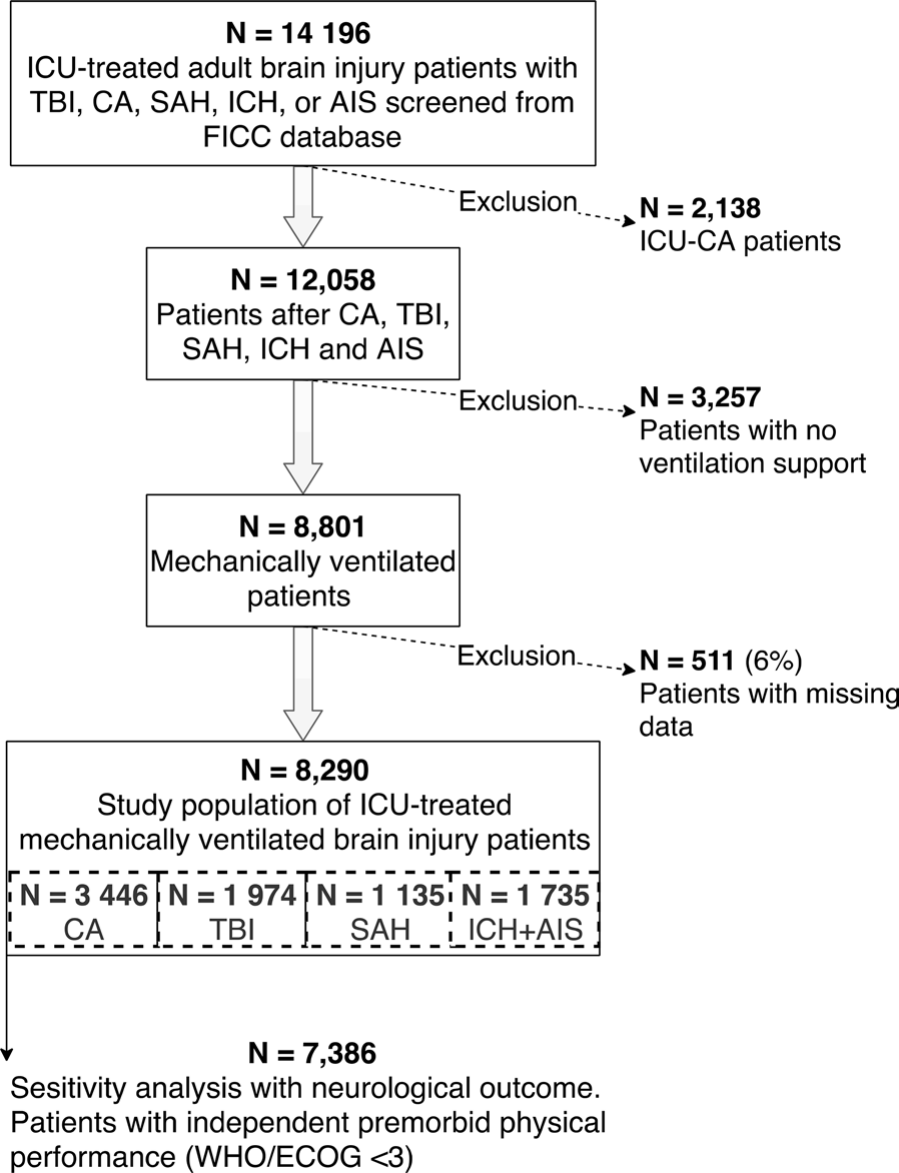
Flowchart of patient inclusion and exclusion. *AIS* acute ischemic stroke, *CA* cardiac arrest, *FICC* Finnish Intensive Care Consortium, *ICH* intracranial hemorrhage, *SAH* subarachnoid hemorrhage, *TBI* traumatic brain injury, *WHO/ECOG* (the World Health Organization/Eastern Co-operative Oncology) performance status score < 3 determined patient as independent.

The FICC database included between 2003 and 2013 14,196 adult ICU-treated patients with traumatic or hypoxic ischemic brain injury. We excluded 2138 patients with CA during intensive care from the study. After excluding patients with no ventilation assistance and patients with missing data, the study sample included 8290 patients (Fig. 1). Frequencies by admission diagnoses were 1974 (24%) with TBI, 3446 (41%) with CA, 1135 (14%) with SAH, and 1735 (21%) with AIS or ICH. The median age was 61, and 2571 (31%) of the study population were female. Altogether, 3912 (47%) patients died within a year of the insult. Regarding survivors, 2704 (62%) survived as independent and 1674 (38%) remained permanently disabled. According to our threshold for hyperoxemia (PaO_2_ > 18.3 kPa), 836 (10%) patients were hyperoxemic, and 5340 (64%) received some vasoactive medication. The baseline characteristics for all patients and patients by diagnosis groups are presented in Table 1 (*found at the end of the manuscript*). Additionally, we determined PaO_2_ and MAP values medians and interquartile ranges by admission year to confirm comparability through study period (2003 to 2013), see results from Additional Table 1.

**Table 1:** Baseline characteristics of study population in whole study sample and in different brain injury populations

	All	TBI	CA	SAH	ICH + AIS
Number of patients (%)	8290	1974 (24)	3446 (41)	1135 (14)	1735 (21)
Age, median [IQR]	61 [50–71]	55 [40–67]	64 [56–73]	57 [49–66]	61 [51–70]
Gender (female), *n* (%)	2571 (31)	444 (23)	906 (26)	642 (57)	579 (33)
Premorbid physical performance independent in self-care (yes), *n* (%)	7386 (89)	1802 (91)	2908 (84)	1097 (97)	1579 (91)
PaO_2_ (kPa), median [IQR]	11.5 [9.6–14.4]	12.8 [10.4–15.9]	10.8 [9–13.5]	11.3 [9.6–14]	11.6 [9.9–14.5]
PaO_2_ groups					
Hypoxemia (< 8.20 kPa), *n* (%)	873 (10.5)	113 (6)	558 (16)	84 (7)	118 (7)
Normoxemia (8.2–18.3 kPa), *n* (%)	6581 (79.4)	1573 (80)	2606 (76)	948 (84)	1454 (84)
Hyperoxemia (> 18.30 kPa), *n* (%)	836 (10.1)	288 (14)	282 (8)	103 (9)	163 (9)
MAP, lowest during 24 h (mmHg), median [IQR]	63 [56–71]	66 [58–73]	61 [53–67]	65 [59–71]	66 [59–76]
MAP tertiles, lowest during 24 h					
< 60 mmHg, *n* (%)	2902 (35)	546 (28)	1557 (45)	327 (29)	472 (27)
60–68 mmHg, *n* (%)	2774 (33)	626 (32)	1217 (35)	431 (38)	500 (29)
> 68 mmHg, n (%)	2614 (32)	802 (40)	672 (20)	377 (33)	763 (44)
ICP monitored, during 24 h *n* (%)	1497 (18)	680 (34)	51 (2)	402 (35)	364 (21)
Vasoactive use, any during 24 h, *n* (%)	5340 (64)	989 (50)	2683 (78)	748 (66)	920 (53)
Glasgow Coma scale at admission, median [IQR]	6 [3–11]	6 [4–11]	5 [3–12]	8 [4–13]	6 [3–10]
Modified SAPS II score^a^, median [IQR]	26 [11–33]	19 [10–30]	30 [15–37]	15 [17–29]	26 [11–31]
Outcome					
Dead at 1 year, *n* (%)	3912 (47)	606 (31)	1971 (57)	419 (37)	916 (53)
Alive but disabled^b^ at 1 year, *n* (%)	1674 (20)	586 (29)	444 (13)	246 (22)	398 (23)
Alive and independent in self-care at 1 year, *n* (%)	2704 (33)	782 (40)	1031 (30)	470 (41)	421 (24)

*AIS* acute ischemic stroke, *CA* cardiac arrest, *GCS* Glasgow Coma Scale, *ICH* intracranial hemorrhage, *IQR* interquartile range, *MAP* mean arterial pressure, *SAH* subarachnoid hemorrhage, *SAPS II* Simplified Acute Physiology Score II, *TBI* traumatic brain injury

^a^SAPS II score excluding point for age, admission type, oxygenation, and systolic blood pressure

^b^disability was determined if a patient was granted a permanent disability allowance 1 year after admission

### Mortality

In the multivariable regression analyses, PaO_2_ was not an independent predictor of 1-year mortality: the OR for hyperoxemia versus normoxemia was 1.16 (95% CI 0.85–1.59) and for hypoxemia versus normoxemia 1.24 (95% CI 0.96–1.61). Higher MAP was associated with lower mortality, OR for MAP 60–68 mmHg versus MAP < 60 mmHg was 0.73 (95% CI 0.64–0.84) and OR for MAP > 68 mmHg versus MAP < 60 mmHg 0.80 (95% CI 0.69–0.92). Additionally, higher age, dependent premorbid physical performance, and more severe disease (higher modified SAPS II score) were independent predictors of higher mortality (Table 2]. There were no significant interactions between groups of PaO_2_ and MAP (the interaction term PaO_2_*MAP was nonsignificant). See Table 2 for all multivariable analysis results of mortality. In the sensitivity analyses, when testing possible subgroup effect in the different diagnosis cohorts, the interaction PaO_2_*MAP was not statistically significant in any of the diagnosis cohorts. Further PaO_2_ was not an independent predictor of mortality in any of the diagnosis cohorts. MAP < 60 mmHg was associated with higher mortality compared to MAP 60–68 mmHg in all other admission diagnosis cohorts except for ICH + AIS. More severe disease and higher age predicted higher mortality in every diagnosis cohort. All results for the sensitivity analyses in disease cohorts are found in Additional Table 2. Results with 90-day mortality were consistent with 1-year mortality (Additional Table 3).

**Table 2:** Adjusted analysis of 1-year mortality

Predictor (*reference category*)	Odds ratio (95% CI)	*p* value
Age	1.03 (1.03–1.04)	< 0.001
Admission diagnosis		
TBI	1 (*reference*)	
CA	1.53 (1.32–1.78)	< 0.001
SAH	1.61 (1.35–1.92)	< 0.001
ICH + AIS	2.3 (1.98–2.68)	< 0.001
Premorbid physical performance independent in self-care (yes)	0.53 (0.45–0.64)	< 0.001
PaO_2_-group		
Normoxemia 8.2–18.3 kPa	1 (*reference*)	
Hyperoxemia > 18.3 kPa	1.16 (0.85–1.59)	0.34
Hypoxemia < 8.2 kPa	1.24 (0.96–1.61)	0.10
MAP tertiles, lowest in 24 h		
< 60 mmHg	1 (*reference*)	
60–68 mmHg	0.73 (0.64–0.84)	< 0.001
> 68 mmHg	0.80 (0.69–0.92)	0.002
Interaction PaO_2_-group * MAP tertiles		
Normoxemia * MAP < 60 mmHg	1 (*reference*)	
Hyperoxemia * MAP 60–68 mmHg	0.76 (0.49–1.16)	0.20
Hypoxemia * MAP 60–68 mmHg	0.83 (0.57–1.22)	0.35
Hyperoxemia * MAP > 68 mmHg	1.03 (0.67–1.57)	0.90
Hypoxemia * MAP > 68 mmHg	1.33 (0.86–2.06)	0.20
Vasoactive, any (yes)	1.08 (0.96–1.21)	0.20
Intracranial pressure monitored (yes)	0.92 (0.79–1.06)	0.25
Modified SAPS II score^a^	1.08 (1.08–1.09)	< 0.001

Area under the receiver operating characteristic curve 0.80 (95% CI 0.79–0.81)

Hosmer–Lemeshow Ĉ goodness-of-fit *p* < 0.05

*AIS* acute ischemic stroke*, CA* cardiac arrest*, CI* confidence interval, *ICH* intracranial hemorrhage*, modified SAPS II* (Simplified Acute Physiology Score II excluding points for age, oxygenation, systolic blood pressure, and type of admission)*, SAH* subarachnoid hemorrhage, *TBI* traumatic brain injury

^a^SAPS II score excluding point for age, admission type, oxygenation, and systolic blood pressure

**Fig. 2:**
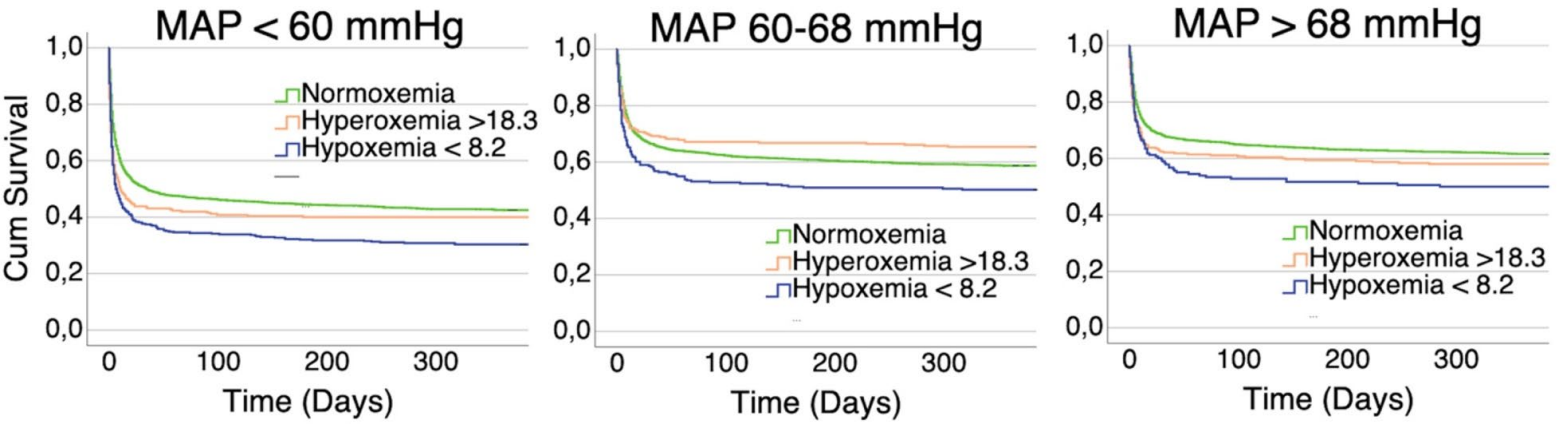
Kaplan–Meier analysis of 1-year mortality between oxygen groups with different mean arterial pressure (*MAP*) levels. One-year mortality was roughly equal between normoxemia and hyperoxemia groups regardless of MAP level. Mortality was highest with hypoxemic patients in every MAP level, and patients with MAP < 60 mmHg had higher mortality compared to higher MAP levels. Logrank p-value with all three analyses < 0.001

The Kaplan–Meier analysis was used to compare mortality in different oxygen groups between MAP tertiles. One-year mortality was highest in the hypoxemia group in all MAP tertiles: 294 (70%) of the hypoxemic cases with MAP < 60 mmHg, 136 (50%) cases with MAP 60–68 mmHg, and 89 (50%) cases with MAP > 68 mmHg were dead at 1 year after the insult. Mortality was roughly equal between normoxemia and hyperoxemia groups in all MAP tertiles, although patients with MAP < 60 mmHg had overall higher mortality compared to MAP 60–68 and > 68 mmHg. The Kaplan–Meier results were statistically significant: the Logrank *p* value was < 0.001 for the analysis in all hemodynamic conditions (Fig. 2).

### Functional Outcome

After exclusion of all patients being disabled prior to admission, 7386 patients remained in the analysis with the functional outcome. Again, higher age and more severe disease predicted lower probability of independent functional outcome. PaO_2_ was not independently associated with the functional outcome. The middle MAP (60–68 mmHg) predicted higher probability of independent functional outcome compared to MAP < 60 mmHg. The interaction term PaO2*MAP was not statistically significant. Results for multivariable analysis with the functional outcome are found from Additional Table 4.

### Locally Weighted Scatterplot Smoothing Curves

**Fig. 3:**
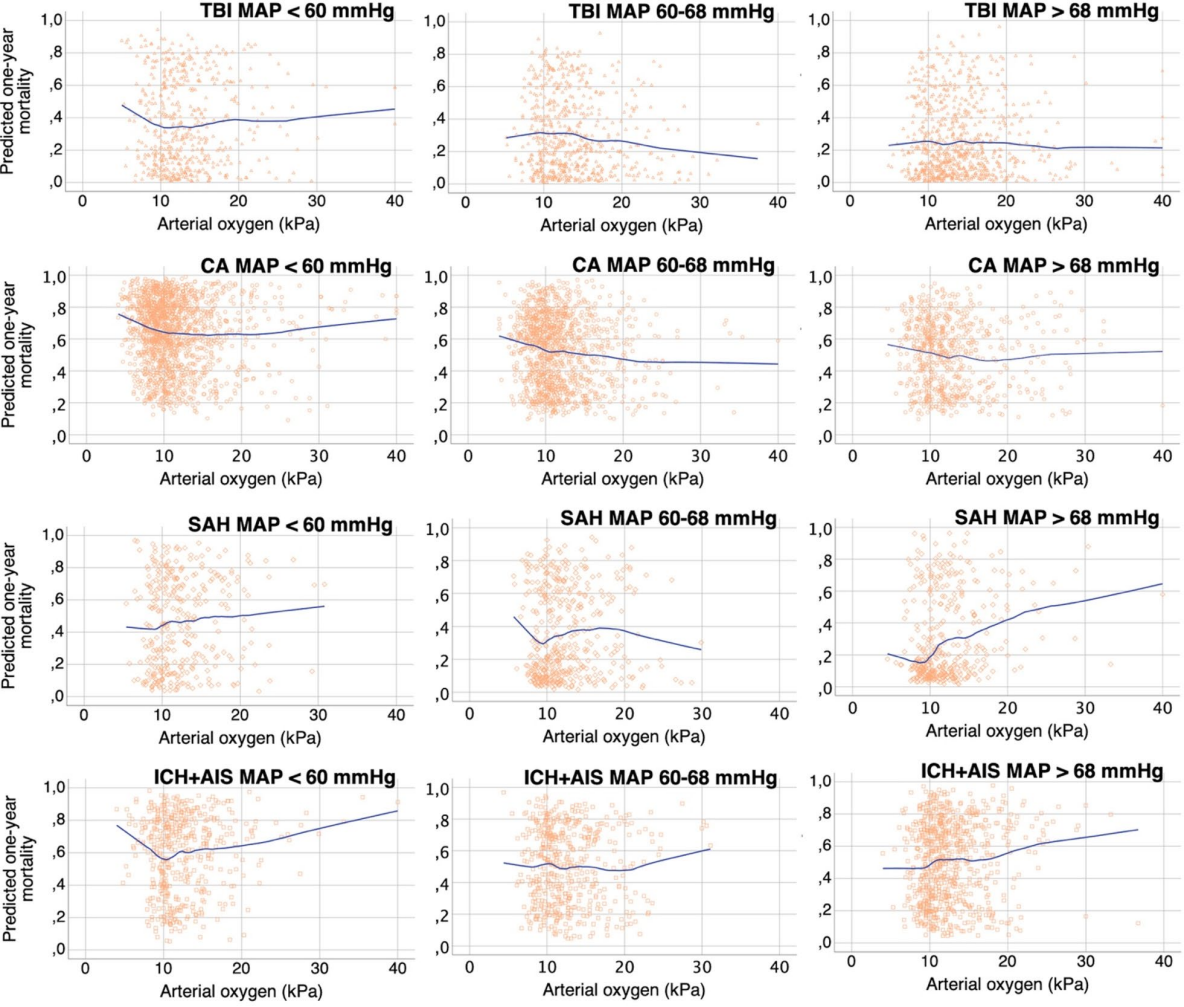
Relationship between arterial partial pressure of oxygen and predicted 1-year mortality with different MAP levels. Locally weighted scatterplot smoothing (Loess) curve visualizing the relationship between arterial oxygen (PaO_2_) and predicted 1-year mortality with different values of mean arterial pressure (MAP). There is a minor trend of higher mortality with low PaO_2_ (about < 10 kPa). The relationship between PaO_2_ and predicted 1-year mortality does not markedly change through MAP levels. Predicted 1-year mortality was calculated separately in every diagnosis cohort and MAP-group with logistic regression analysis with the following parameters: age, preadmission status independent in self-care, intracranial pressure measured, any vasoactive used, modified SAPS II score (excluding points for age, oxygenation, admission type and systolic blood pressure), and TBI Marshall classification in TBI. The performance of predicting the actual 1-year mortality was good in every subgroup; in all subgroups the area under the curve (AUC) > 0.75

The relationships between PaO_2_ and the predicted probability of 1-year mortality were visualized with Loess curves in every diagnosis cohort separately for different MAP tertiles (Fig. 3). Sixteen PaO_2_ values > 40 kPa were coded as 40 kPa in the analyses. There were no dramatic differences in the relationships between PaO_2_ and predicted mortality between MAP tertiles (similar curve shapes) in any diagnosis cohorts. Thus, the association between PaO_2_ and mortality is constant regardless of MAP. Yet, within all diagnosis cohorts, there was a minor trend toward higher mortality with lower PaO_2_ (PaO_2_ about < 10 kPa). The effect was most visible in the lowest MAP (< 60 mmHg). Overall in SAH and ICH + AIS cohorts the predicted mortality was slightly higher with hyperoxemia compared to normoxemia but the small amount of cases with hyperoxemic PaO_2_ values makes the association weak.

## Discussion

### Key Findings

In this large retrospective cohort analysis of patients with various type of brain injury, we found no association between the presence of hyperoxemia and improved long-term outcome in patients with low MAP during the first 24 h. Low MAP (< 60 mmHg) was an independent predictor of mortality, but the effect was not alleviated in patients with higher oxygen. Our additional analysis focusing on long-term functional outcome as a surrogate marker of neurological recovery resulted in similar findings. The current analysis, acknowledging its retrospective design and clear limitations to show causality, nonetheless, does not support the hypothesis that higher blood oxygen content in neurocritically ill patients could compensate for the harmful effects of impaired systemic circulation and low MAP.

### Relationship with Previous Studies

Multiple studies have been conducted on the associations between oxygen and outcome in patients with different types of brain injury. In TBI patients, hyperoxemia has been associated with worse survival with extreme and better survival with moderate hyperoxemia [3, 9, 22, 23]. However, in a recent meta-analysis of hyperoxemia after TBI, including six epidemiological studies, hyperoxemia did not influence mortality [24]. Extreme hyperoxemia after SAH has been found to increase mortality and is claimed to induce or exacerbate delayed cerebral ischemia [25–27]. ICH and AIS are regularly studied together: one study found an association between hyperoxemia and higher mortality, whereas another did not [6, 28]. One meta-analysis studied hyperoxemia and mortality after SAH, ICH, or AIS and concluded that when all three cohorts were inspected together, no association existed between high oxygen and outcome. Additionally, another meta-analysis inspected SAH and AIS in separate cohorts and hyperoxemia was associated with higher mortality in AIS but not in SAH patients [24, 29]. In a large meta-analysis of 25 randomized controlled trials with 16 037 acutely ill heterogenous ICU-treated patients, Chu et al. compared conservative versus liberal oxygen therapy [30]. In individual studies, the results were heterogenic, but the combined effect suggested benefit with conservative compared to liberal oxygen therapy. None of the previous studies have in any detailed fashion studied the differential association between the oxygen and MAP with outcome. Guidelines on oxygenation and blood pressure after acute neurologic injury are debatable, and lack high-quality evidence base. For TBI specific PaO_2_ or SpO_2_ targets are not given, only monitoring of jugular venous saturation and PbO_2_ is recommended without specific targets [31]. For ischemic stroke SpO_2_ > 94% and for CA SpO_2_ 94–98% are recommended and routine supplemental oxygen advised not to be used if SpO_2_ -target is met [32, 33]. Loose blood pressure targets are specified after TBI (systolic blood pressure, SBP > 100 mmHg), CA (MAP > 65 mmHg and SBP > 90 mmHg), and SAH (< 160 mmHg) [33–35].

It is well recognized that aerobic metabolism is reduced in injured brain areas and oxygen therapy has been claimed to restore the brain aerobic function [7, 36, 37]. An increase in PaO_2_ enhances oxygen diffusion and leads to higher brain tissue oxygen (PbO_2_) even in the setting of normal cerebral perfusion pressure and MAP [12]. High PaO_2_ has also been shown to cause vasoconstriction which can reduce brain edema and enhance brain perfusion [36, 38–41]. Increasing PaO_2_ could be of theoretical benefit especially if adequate cerebral perfusion pressure is not achieved and, indeed, the use of 100% oxygen in the severely shocked patient (i.e., during severe hemorrhage) is common practice, even though conclusive evidence in humans is lacking. Early onset NBO therapy after AIS has been claimed to preserve the hypoperfused core infarction area and penumbra [42, 43]. In a large randomized clinical trial, routine low-dose supplemental oxygen (2–3 l/min) administered in the ICU continuously or at night only for the initial 72 h after AIS did not improve outcome, but NBO therapy was concluded to be safe [11]. It is, however, largely unclear if the injured brain can utilize supranormal oxygen levels [12, 44]. Furthermore, by far the known hyperoxemia mediated changes to cerebral vascular tension, perfusion, and tissue are partly opposing and true net change in oxygen delivery to the brain site in need can remain near zero [36, 38, 40, 45].

### Clinical Implications of Study Findings

We did not find any clear association between oxygen level and MAP during the first 24 h and outcome in patients with various types of brain injury. The “optimum” PaO_2_ during the initial ICU treatment after brain injury, despite the hemodynamic conditions, seems to be near the physiological level or slightly but not greatly above it, which supports the findings of several studies indicating better outcomes of moderate but not extreme hyperoxemia exposure after brain injury [4, 6, 9, 10, 22]. Some clinical studies have been conducted in patients treated following cardiac arrest, but thus far the evidence is conflicting and larger trials are needed in neurocritically ill patients.

### Strengths and Limitations

We acknowledge several strengths of our study. Our sample of brain injury patients is large and includes patients from several diagnosis cohorts with differences in initial disease pathophysiology but similarities in injury responses after the insult [46]. In addition, patients were treated at multiple centers in Finland, increasing validity. We used data from the Social Insurance Institution of Finland (Kela) database, including data on the disability allowance of study participants 1 year after admission, as a surrogate marker for long-term functional outcome. All Finnish residents belong to the social insurance system and data on possible dates of death were available from Finnish population register for all patients; hence, loss of follow-up in this study is nonexistent.

However, we also recognize limitations. Due to limitations in the used dataset, we only had a single PaO_2_ value for our analyses (the value recorded in connection with the lowest PaO_2_/FiO_2_ ratio during the first 24 h in the ICU) and could not inspect actual oxygen burden over time. However, we have confirmed in a previous study that in mechanically ventilated TBI patients the PaO_2_ used in the current study correlates well with the mean PaO_2_ of the first 24 h in the ICU [9]. Further on, this same strategy of categorizing oxygenation status with just one PaO_2_ value has also been used in scientific studies focusing on CA patients [47]. In addition, our threshold for hyperoxemia (> 18.3 kPa) was set relatively low compared to some previous studies which may mask some of the effects of severe hyperoxemia [4, 22, 23]. However, the used PaO_2_ value is likely the lowest measured during the first 24 h of ICU care, indicating that other PaO_2_ measurements during the 24-h period were higher. For some patients, this study can misleadingly group a patient as hypoxemic based on only one temporal low PaO_2_ value. Also, we grouped ICH and AIS patients together due to the small number of participants in each group; a similar grouping is been undertaken in other studies [6]. We only had the lowest MAP value of the first 24 h in the ICU and therefore cannot draw conclusions about the duration of hypotension and its effects. Hence, used MAP levels should not be seen as absolute indicators for actual blood pressure levels over time but as surrogate markers of the hemodynamic status over the first 24 h. There is, however, evidence that the lowest MAP measured during the initial hours in the ICU is associated with outcome in patients after CA as well as in patients with circulatory shock related to sepsis and acute pancreatitis [48–50]. Last, at the time of the data collection, arterial CO_2_ was not included in the FICC database, and we could not consider it as a confounding factor in this study although it to a great degree impacts on cerebral perfusion pressure.

## Conclusions

Within different brain injury populations during the initial 24 h of ICU treatment, we found no signs of benefit from hyperoxemia exposure for survival or 1-year functional outcome in the presence of low compared to normal or high mean arterial pressure. Recognizing the retrospective nature of our study, the results do not indicate a need to tailor oxygen used based on MAP level as a surrogate marker of cerebral perfusion in neurocritically ill patients.

## Electronic supplementary material

Below is the link to the electronic supplementary material.

Supplementary material 1 (DOCX 27 kb)
