# Supplementary material for: The Association Between Arterial Oxygen Level and Outcome in Neurocritically Ill Patients is not Affected by Blood Pressure
Source: Neurocrit Care. 2021 Jan 5;34(2):413–22. doi: 10.1007/s12028-020-01178-w (PMC8128839; doi:10.1007/s12028-020-01178-w)
Supplement: Supplementary file 1 — Supplementary material 1 (DOCX 27 kb) [file 12028_2020_1178_MOESM1_ESM.docx]

**Additional Table 1** One-year mortality, PaO_2_, and MAP by admission year through the study period

| **Admission year** | **Dead at one year, n(%)** | **PaO_2_ kPa, median/[IQR]** | **MAP mmHg, median/[IQR]** |
| --- | --- | --- | --- |
| 2003 | 295 (50) | 11.5 [9.6-14.3] | 64 [56-72] |
| 2004 | 344 (54) | 11.6 [9.7-14.3] | 65 [55-73] |
| 2005 | 363 (49) | 11.3 [9.4-14.3] | 63 [54-71] |
| 2006 | 410 (50) | 11.3 [9.6-14.3] | 64 [56-72] |
| 2007 | 419 (50) | 11.2 [9.2-14.2] | 64 [57-71] |
| 2008 | 339 (45) | 11.1 [9.3-13.7] | 63 [56-71] |
| 2009 | 299 (43) | 11.5 [9.5-15.2] | 65 [58-72] |
| 2010 | 356 (46) | 11.8 [9.7-15.1] | 63 [57-70] |
| 2011 | 369 (46) | 11.7 [9.6-14.8] | 63 [57-69] |
| 2012 | 395 (44) | 11.9 [9.9-14.9] | 62 [56-69] |
| 2013 | 323 (44) | 11.3 [9.6-14.1] | 63 [56-68] |
| **p-value** | Chi-Square <0.001 | Kruskal-Wallis 0.001 | Kruskal-Wallis <0.001 |
| *IQR interquartile range, PaO_2_ partial pressure of arterial oxygen, MAP mean arterial pressure,* | | | |

**Additional Table 2** Adjusted analysis of 1-year mortality in different brain injury populations

| **Predictor (*reference category*)** | **Odds ratio (95% confidence interval)** | | | |
| --- | --- | --- | --- | --- |
|  | **TBI** | **CA** | **SAH** | **ICH+AIS** |
| Age | 1.05 (1.04-1.05) * | 1.02 (1.02-1.03) * | 1.04 (1.03-1.06) * | 1.04 (1.03-1.05) * |
| Preadmission status independent in self-care (yes) | 0.61 (0.41-0.91) * | 1.02 (1.02-1.03) * | 0.42 (0.19-0.95) * | 0.74 (0.50-1.10) |
| PaO_2_-group (*normoxemia, 8.2-18.3 kPa*) | *1 (reference)* | *1 (reference)* | *1 (reference)* | *1 (reference)* |
| hyperoxemia (PaO_2_ >18.3 kPa) | 0.89 (0.47-1.71) | 1.21 (0.76-1.93) | 0.94 (0.39-2.27) | 1.40 (0.63-3.10) |
| hypoxemia (PaO_2_ < 8.20 kPa) | 1.78 (0.81-3.89) | 1.17 (0.86-1.58) | 0.56 (0.19-1.69) | 2.57 (0.95-6.99) |
| MAP tertiles, worst in 24 h  (*MAP < 60 mmHg*) | *1 (reference)* | *1 (reference)* | *1 (reference)* | *1 (reference)* |
| MAP 60-68 mmHg | 0.71 (0.51-1.00) * | 0.76 (0.62-0.92) * | 0.58 (0.39-0.86) * | 0.76 (0.55-1.05) |
| MAP > 68 mmHg | 0.80 (0.58-1.12) | 0.76 (0.60-0.97) * | 0.75 (0.49-1.15) | 0.81 (0.60-1.09) |
| Interaction PaO_2_ * MAP tertiles | *1 (reference)* | *1 (reference)* | *1 (reference)* | *1 (reference)* |
| Hyperoxemia * MAP 60-68 mmHg | 0.85 (0.34-2.12) | 0.47 (0.24-0.92) * | 1.44 (0.44-4.71) | 1.08 (0.39-3.01) |
| Hypoxemia * MAP 60-68 mmHg | 0.78 (0.22-2.71) | 0.90 (0.57-1.44) | 1.89 (0.46-7.67) | 0.38 (0.11-1.30) |
| Hyperoxemia * MAP >68 mmHg | 1.25 (0.55-2.84) | 0.81 (0.40-1.66) | 2.14 (0.59-7.71) | 0.89 (0.33-2.42) |
| Hypoxemia * MAP >68 mmHg | 1.04 (0.34-3.26) | 1.01 (0.54-1.87) | 4.13 (0.95-18.04) | 0.90 (0.26-3.11) |
| Vasoactive, any (yes) | 1.58 (1.23-2.02) * | 0.84 (0.69-1.01) | 1.01 (0.72-1.41) | 0.91 (0.72-1.15) |
| Intracranial pressure measured | 0.94 (0.72-1.23) | 1.37 (0.71-2.64) | 0.78 (0.57-1.06) | 0.77 (0.58-1.01) |
| Modified SAPS II score^a^ | 1.10 (1.08-1.11) * | 1.06 (1.06-1.07) * | 1.12 (1.10-1.13) * | 1.09 (1.08-1.10) * |
| TBI Marshall class. (*I*) | *1 (reference)* | *Not applicable* | | |
| II | 0.89 (0.51-1.55) |  |  |  |
| III | 2.09 (1.11-3.91) * |  |  |  |
| IV | 3.25 (1.22-8.63) * |  |  |  |
| V | 1.84 (1.10-3.07) * |  |  |  |
| *** statistically significant result (p < 0.05), ^a^SAPS II score excluding point for age, admission type, oxygenation and systolic blood pressure  *TBI* traumatic brain injury*, CA* cardiac arrest*, SAH* subarachnoid hemorrhage*, ICH* intracranial haemorrhage*, AIS* acute ischemic stroke*, MAP* mean arterial pressure, *SAPS II* Simplified Acute Physiology Score II | | | | |

| **Predictor (*reference category*)** | **Odds ratio (95% CI)** | **p-value** |
| --- | --- | --- |
| Age | 1.03 (1.02-1.03) | <0.001 |
| Admission diagnose (*TBI*) | 1 (*reference*) | <0.001 |
| CA | 1.60 (1.34-1.81) | <0.001 |
| SAH | 1.75 (1.46-2.10) | <0.001 |
| ICH + AIS | 2.38 (2.04-2.78) | <0.001 |
| Premorbid physical performance independent in self-care (yes) | 0.63 (0.53-0.75) | <0.001 |
| PaO_2_-group (Normoxemia 8.2-18.3 kPa) | 1 (*reference*) | 0.10 |
| Hyperoxemia > 18.3 kPa | 1.31 (0.96-1.78) | 0.09 |
| Hypoxemia < 8.2 kPa | 1.31 (0.96-1.78) | 0.15 |
| MAP tertiles, worst in 24h (MAP *< 60 mmHg*) | 1 (*reference*) | <0.001 |
| MAP 60-68 mmHg | 0.73 (0.64-0.84) | <0.001 |
| MAP > 68 mmHg | 0.78 (0.68-0.91) | 0.001 |
| interaction PaO_2_-group * MAP tertiles (*normoxemia*MAP < 60*) | 1 (*reference*) | 0.21 |
| Hyperoxemia * MAP 60-68 | 0.74 (0.49-1.14) | 0.17 |
| Hypoxemia * MAP 60-68 | 0.93 (0.64-1.37) | 0.72 |
| Hyperoxemia * MAP > 68 | 0.94 (0.62-1.44) | 0.79 |
| Hypoxemia * MAP > 68 | 1.44 (0.94-2.22) | 0.10 |
| Vasoactive, any (yes) | 1.09 (0.97-1.22) | 0.16 |
| intracranial pressure measured (yes) | 0.87 (0.75-1.01) | 0.07 |
| Modified SAPS II score^a^ | 1.08 (1.08-1.09) | <0.001 |
| ^a^SAPS II score excluding point for age, admission type, oxygenation, and systolic blood pressure | | |
| *CI* confidence interval, *TBI* traumatic brain injury*, CA* cardiac arrest*, SAH* subarachnoid hemorrhage*, ICH* intracranial hemorrhage*, AIS* acute ischemic stroke*, modified SAPS II* Simplified Acute Physiology Score II excluding points for age, oxygenation, systolic blood pressure and type of admission | | |

**Additional Table 3** Adjusted analysis of 90-day mortality

**Additional Table 4** Adjusted analysis of functional outcome (independent functional status at one year was determined as good outcome and disability or death as poor outcome)

| **Predictor (*reference category*)** | **OR for good outcome (95% CI)** | **p-value** |
| --- | --- | --- |
| Age | 0.97 (0.97-0.97) | <0.001 |
| Admission diagnose (*TBI*) | 1 (*reference*) | <0.001 |
| CA | 1.31 (1.12-1.53) | 0.001 |
| SAH | 0.98 (0.83-1.16) | 0.813 |
| ICH + AIS | 0.59 (0.50-0.70) | <0.001 |
| PaO_2_-group (*Normoxemia 8.2-18.3 kPa*) | 1 (*reference*) | 0.52 |
| Hyperoxemia > 18.3 kPa | 1.11 (0.79-1.55) | 0.56 |
| Hypoxemia < 8.2 kPa | 0.87 (0.65-1.17) | 0.36 |
| MAP tertiles, worst in 24h (*MAP <60 mmHg*) | 1 (*reference*) | <0.001 |
| MAP 60-68 mmHg | 1.31 (1.13-1.52) | <0.001 |
| MAP >68 mmHg | 1.02 (0.87-1.18) | 0.84 |
| Interaction PaO_2_-group * MAP tertiles (*normoxemia* MAP <60 mmHg*) | 1 (*reference*) | 0.63 |
| Hyperoxemia * MAP 60-68 mmHg | 1.17 (0.75-1.83) | 0.48 |
| Hypoxemia * MAP 60-68 mmHg | 0.90 (0.58-1.38) | 0.61 |
| Hyperoxemia * MAP >68 mmHg | 0.86 (0.55-1.35) | 0.52 |
| Hypoxemia * MAP >68 mmHg | 0.87 (0.54-1.41) | 0.57 |
| Vasoactive, any, yes | 1.11 (0.99-1.25) | 0.09 |
| Intracranial pressure measured, yes | 1.00 (0.86-1.15) | 0.96 |
| Modified SAPS II score^a^ | 0.94 (0.93-0.94) | <0.001 |
| ^a^SAPS II score excluding point for age, admission type, oxygenation, and systolic blood pressure  *OR* odds ratio, *CI* confidence interval, *TBI* traumatic brain injury*, CA* cardiac arrest*, SAH* subarachnoid hemorrhage*, ICH* intracranial hemorrhage*, AIS* acute ischemic stroke*, modified SAPS II* Simplified Acute Physiology Score II excluding points for age, oxygenation, systolic blood pressure and type of admission | | |
| Area under the receiver operating characteristics curve 0.75 (0.74–0.76) | | |
| Hosmer-Lemeshow Ĉ goodness-of-fit p = 0.83 | | |
